# Atrial fibrosis progression in patients with Atrial Fibrillation

**DOI:** 10.1186/1532-429X-18-S1-P149

**Published:** 2016-01-27

**Authors:** Robert B White, Mohamed Labedi, Jordan King, Christina F Pacchia, Kara Johnson, Gagandeep Kaur, Joshua Cates, Alan Morris, Nassir F Marrouche

**Affiliations:** grid.417538.cCardiovascular Center, University of Utah Hospital, Providence, UT USA

## Background

Fibrosis is a hallmark of arrhythmogenic structural remodeling in patients with Atrial Fibrillation (AF). Recently, the degree of atrial fibrotic changes has been correlated with stroke and poor treatment outcome in patients with AF. In this study, we report temporal behavior of atrial fibrosis.

## Methods

Eighty-eight patients (58% male, mean age 60.4+/-14.7) with AF who underwent late gadolinium enhancement MRI (LGE-MRI) to assess the degree of atrial tissue fibrotic changes were included in this study. All patients underwent at least 2 or more LGE-MRI separated by more than 3 months of follow up. Progression of fibrosis was defined as an increase in fibrosis area by more than 5% (Figure 1). Demographic patient data as well as comorbidities and medications were collected from chart revisions.

## Results

A total of 32 patients (36.4%) had hypertension, 21 (23.9%) with hyperlipidemia, 9 (10.2%) with diabetes, 6 (6.8%) patients had with a history of tobacco abuse and 10 (11.4%) with coronary artery disease. A total of 38 (43.2%) patients were on statin, 25 (28.4%) were on angiotensin converting enzyme inhibitor, 36 (40.9%) were on a beta-°©-blocker, 31 (35.2%) were on anticoagulant, and 13 (14.8%) were on antiarrhythmic medication. Subsequent follow-°©-up MRIs were performe on 32 (36%) patients that exhibited progression of atrial fibrosis.

Using univariate logistic regression models we were not able to identify significant predictors of fibrosis progression. The majority of patients (64%) did not reveal any significant changes from the initial quality and quantity of atrial fibrosis at 1 year MRI follow-up.

## Conclusions

Atrial fibrosis quantified by LGE-MRI seems to be stable in a majority of AF patients at one-year follow-up. Approximately a third of patients evaluated exhibit progression of atrial fibrotic disease. A means to alleviate progression in this population would improve surrogate outcome.

